# Assessing the impact of Ghana's geology on gold mining using geospatial techniques

**DOI:** 10.1016/j.heliyon.2025.e42921

**Published:** 2025-02-21

**Authors:** Aaron Tettey Tetteh, Yakubu Issaka, Philomena Dodoo, Bernice Atugba, Albert Tengnibuor

**Affiliations:** a School of Mines and Built Environment, University of Energy and Natural Resources, Sunyani, Ghana; b University of Mines and Technology, Tarkwa, Ghana

**Keywords:** Gold-bearing rocks, Tarkwaian, Birimian sediments, Geospatial techniques, AngloGold Ashanti

## Abstract

As the largest gold producer in Africa, Ghana's spatial distribution of gold-bearing formations is still largely unexamined on a national scale. Previous studies have largely focused on specific mining regions, neglecting a holistic geospatial assessment of gold-bearing rocks across the country. This study employs geospatial techniques to evaluate the relationship between Ghana's geological formations and gold mineralization. The results reveal that gold mineralization is primarily associated with Birimian Volcanics, Birimian Sediments, and Tarkwaian rocks, which are distributed across 12 regions, with the highest concentrations in the Bono (18.74 %), Ashanti (17.93 %), Western (17.53 %), and Western North (11.45 %) regions. The total area occupied by gold-bearing formations is 55,723 km^2^ (23.34 %) of Ghana's landmass. Notably, large-scale mining operations such as Newmont Gold Ghana Limited and AngloGold Ashanti predominantly operate within these geological formations, confirming the strong relationship between rock type and gold deposits. This study emphasizes the importance of considering the spatial distribution of gold-bearing formations in land-use planning and future mining investments to optimize resource management and mitigate conflicts between urbanization and mineral exploration.

## Highlights

• Ghana is Africa's top gold producer, with deposits in 12 regions.
• Birimian Sediments cover 65.99 % of Ghana's gold-bearing formations.
• Bono Region has 18.74 % of gold-bearing rocks, leading other regions.
• 23.34 % of Ghana's land hosts gold-bearing rock formations.
• Strategic planning is needed to protect gold zones from urbanization.

## 1 Introduction

Gold mineralization is influenced by many geological characteristics globally. For instance, the ductile shear zone (DSZ) plays a crucial role in the formation process of large-scale gold deposits. In China, the greenstone-granite geological formation located within the Jiaodong Peninsula is characterized by notable gold mineralization, wherein carbonate-sulfide vein-type deposits play a crucial role in enhancing its economic significance [1,2]. Similarly, the Paishanlou gold deposit in Liaoning Province is a notable example of shear zone-controlled mineralization [3]. In North Africa, the Hoggar Shield is recognized for its gold-rich structures [4], while the Yilgarn Shield in Western Australia is a key region for orogenic gold deposits [5]. The Rloltapicuru granite-greenstone belt in Brazil also contains significant gold occurrences [6]. In India, the Hutti-Maski granite-greenstone belt is well known for its association with gold-bearing quartz veins [6]. Understanding these geological settings is crucial, as tectonic evolution plays a significant role in gold mineralization [7]. In Cameroon, primary gold occurrences are associated with quartz veins within the large-scale Central Cameron Shear Zone [8]. The Jiaodong gold deposits in China mostly occur in the Linglong and Guojialing granitoid suites, but no apparent gold mineralization exists in the Weideshan and Laoshan granitoid suites [9,10]. This gave a specific class of rocks bearing gold.

In Ghana, gold mineralization is deposited within Tarkwaian, Birimian Sediments and Birimian Volcanics rocks [[11], [12], [13], [14], [15], [16], [17], [18], [19], [20]].

Gold mineralization in Ghana mainly occurs in two styles namely: mesothermal quartz vein-hosted and associated gold in metavolcanics and metasediments and modified palaeoplacer gold in conglomerates. These styles can be categorized under the Precambrian rocks of the West African Craton, specifically the Birimian Supergroup and the Tarkwaian Group that make up Ghana's mainly southwest to northeast trending Birimian belts [19]. The Eburnean Orogeny is one of the most critical tectonic events that is particularly associated with the formation and deformation of the Birimian Supergroup and the Tarkwaian Group.

The Birimian greenstone belts are associated with the lode gold deposits have formed through hydrothermal processes, where gold-bearing fluids were transported through fault and shear zones, depositing gold in fractures and quartz veins [21]. These deposits are particularly found within the southwestern of Ghana which is one of the richest gold endowed terranes in the West African Craton [22]. Théveniaut & Clarke [23], confirmed that gold is the most important economic element deposited within the Birimian and Tarkwaian rocks. Ghana is the home for gold in Africa, being the largest gold producer in Africa and sixth in the world [24].

Geographic Information System (GIS) has become a key catalyst for analyzing information about the Earth, especially in the past few decades [25]. GIS has gained numerous applications in environmental management, mining, agriculture, health, and many other areas. For instance, geospatial techniques were employed to assess wetlands [26,27]. Subsiquently, Tetteh et al. [28], applied geospatial techniques to assess pluvial flood prone areas in Sunyani, Ghana. Furthermore, Moomen et al. [29], underscores how Asaana and Sadick applied geospatial techniques in 2016 to evaluate the irrigation performance of the Tono irrigation system. Lin et al. [30] applied geospatial techniques for ecological evaluation in Fuzhou City, China, while Bencharef et al. [31] utilized geospatial techniques to perform polymetallic mineralization prospectivity modeling. These studies have proven the capabilities of using GIS in other fields, such as gold mineralization.

A study on the geology drill impact on structures considering Birimian rocks for Bosumtwi in the Ashanti Region, Ghana was done by Boamah & Koeberl, [32]. Osae et al. [17], did a study on ore mineralogy Ashanti gold deposit at Obuasi, Ghana, and established that gold deposits are associated with Tarkwaian and Birimian rocks.

Despite Ghana's status as the largest gold producer in Africa, there exists a conspicuous absence of a comprehensive geospatial assessment of gold-bearing lithological formations at the national scale. Existing studies have primarily focused on specific mining regions such as Tarkwa, Obuasi, and Prestea, without systematically analyzing the spatial distribution of gold-hosting formations across the country. This lack of a holistic geospatial assessment limits decision-making for investors, policymakers, and environmental managers.

Methods involving geospatial analysis, including GIS and Remote Sensing, have demonstrated their efficacy in mineral exploration by delivering spatially detailed information for resource management. Recent advancements in GIS allow for high-precision mapping of geological formations, enabling a more detailed understanding of gold-bearing zones. By integrating these techniques, this study fills the existing research gap by offering a spatially comprehensive analysis of gold mineralization potential across Ghana.

Therefore, this study aims to apply geospatial techniques to assess Ghana's gold-bearing rocks and their impact on gold mining activities. The specific objectives are to: a) Identify and classify the gold-bearing rock formations in Ghana using GIS-based geological mapping, b) Quantify and map the spatial distribution of these formations across Ghana's administrative regions, c) Analyze the correlation between gold-bearing rocks and the locations of large-scale mining operations, and d) Validate findings using ground truth verification via Google Earth Pro and field surveys to ensure accuracy.

(a) What are the key gold-bearing rock formations in Ghana, and how are they spatially distributed? (b) How do the spatial extents of these formations vary across different administrative regions? (c) To what extent does the distribution of gold-bearing rocks influence the locations of active mining operations? (d) How can geospatial techniques enhance the assessment and visualization of Ghana's gold mineralization potential? By addressing these research questions, we identify the gold-bearing rock, including their area and percentages, and the location of mining companies within the study area, providing a vital framework for investors, government, and researchers on development decision-making. Knowledge of the spatial distribution and area of the gold bearing rocks could help researchers, stakeholders and miners to take informed decisions such as impact of gold mining on socio-economic environment in the mining communities.

## 2 Material and methods

### 2.1 Study area

**Fig. 1:**
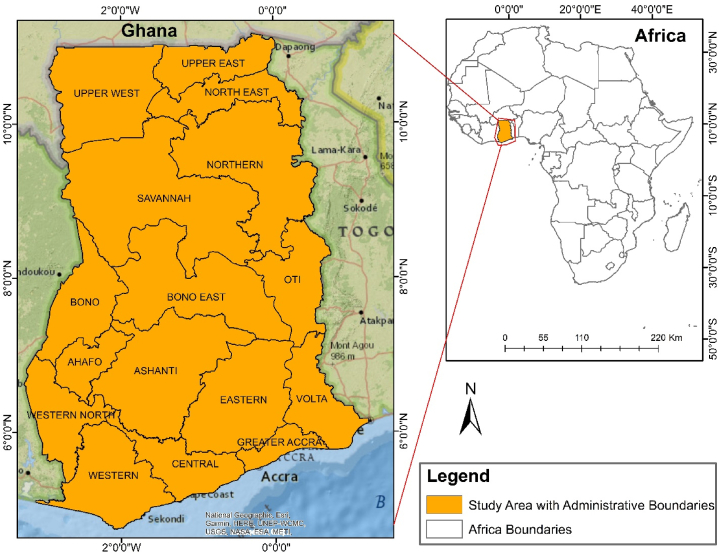
Map of the study area, Ghana, showing its geographical location within Africa and administrative boundaries.

The study area was Ghana, a West African country in the Gulf of Guinea. Its neighbours are Côte d'Ivoire to the west, Burkina Faso, which lies north, and Togo to the east. It is situated between latitudes 06°00ʹ00ʺ N - 10°00ʹ00ʺ N and longitudes 00°00ʹ00ʺ W - 02°00ʹ00ʺ W, covering an area of about 238,540 km^2^ (Fig. 1). Ghana is comparatively low at an average elevation of 190 m above sea level, and the highest elevation (Mount Afadjato) is 880 m [33]. Ghana is a major exporter of gold, timber, industrial diamonds, bauxite, manganese, fish, rubber, hydropower, petroleum, silver, salt, limestone, and cocoa [34]. The geology of Ghana falls within the southeastern portion of the West African Craton, and it is divided into four tectonostratigraphic units, namely, Late Palaeozoic to Mesozoic sedimentary basins, mobile belt (Dahomeyide Belt) situated to the east of the West African Craton, early Proterozoic crystalline rocks collectively known as the Birimian and Tarkwaian rocks with ages ranging between ∼2.31 and 2.06 Ga, and Neo-Proterozoic sedimentary cover called the Voltaian Basin [35,36]. Ghana is rich in mineral resources such as gold, diamond, bauxite, and manganese, which contribute significantly to exports and revenue generation. Regarding gold production, Ghana is the largest producer in Africa and sixth globally [24]. Ghana is divided into sixteen administrative regions, each with its respective capital town [37] and the national capital being Accra. Ghana's population is 30.8 million, according to the 2021 census report [38].

### 2.2 Literature review

Gold mineralization plays a vital role in the global economy, particularly in regions with rich geological structures conducive to the formation and accumulation of gold deposits. Globally, gold mineralization is predominantly found in regions characterized by complex geological structures such as greenstone belts, shear zones, and fault systems [39]. Countries like Canada, Australia, and South Africa are renowned for their gold deposits, which are often associated with ancient metamorphic rocks such as the Archean and Proterozoic greenstone belts. These formations have been the focus of extensive geological and geophysical studies, and the application of advanced geospatial techniques has enabled more accurate exploration. A study conducted by Goldfarb & Groves [40] established that the greenstone belts of Australia, shear zones of India and Brazil, and granitoid suites of China possess gold endowments.

Africa's gold mineralization is strongly associated with its ancient cratonic regions and greenstone belts, which are key geological features across the continent. Notably, the West African Craton, home to the Birimian and Tarkwaian rock formations in countries like Ghana, Mali, and Burkina Faso, is rich in gold deposits [12,41]. These greenstone belts, along with others like the Zimbabwe Craton and the Kaapvaal Craton in South Africa, have undergone extensive tectonic activity, leading to the formation of gold-bearing veins and lodes [42]. The Witwatersrand Basin in South Africa, the largest known gold deposit in the world, is another critical formation, comprising conglomerate layers filled with gold [43]. Other significant gold-bearing regions include the Lufilian Arc in the Democratic Republic of Congo and the Nubian Shield in the northeast, which extends into Egypt and Sudan, making Africa one of the richest continents in terms of gold resources [44]. In Cameroon, primary gold occurrences are associated with quartz veins within the large-scale Central Cameron Shear Zone [8].

Gold production in Ghana has been in existence prior to discovery of the Witwatersrand goldfields of South Africa. The production of gold in Ghana has given to rise to about 8 % in 2023 increasing the overall production of gold to 4 million ounces. This represents a significant proportion of revenue generated in the country. Gold mineralization in Ghana mainly occurs in two styles namely: mesothermal quartz vein-hosted and associated gold in metavolcanics and metasediments and modified palaeoplacer gold in conglomerates. These styles can be categorized under the Precambrian rocks of the West African Craton, specifically the Birimian Supergroup and the Tarkwaian Group that make up Ghana's mainly southwest to northeast trending Birimian belts [19]. The Eburnean Orogeny is one of the most critical tectonic events that is particularly associated with the formation and deformation of the Birimian Supergroup and the Tarkwaian Group.

The Birimian greenstone belts are associated with the lode gold deposits have formed through hydrothermal processes, where gold-bearing fluids were transported through fault and shear zones, depositing gold in fractures and quartz veins [21]. These deposits are particularly found within the southwestern of Ghana which is one of the richest gold endowed terranes in the West African Craton [22].

Gold mining in Ghana has significantly contributed to the country's economic development, making it the largest gold producer in Africa and sixth in the world [24]. The spatial distribution of gold-bearing formations and the geological characteristics of the country play a crucial role in determining the locations of gold deposits. Ghana's geology is dominated by the Birimian and Tarkwaian rock formations, which have long been identified as the primary gold-bearing units in the country [11,12,19]. These formations are part of the West African Craton and are geologically significant for hosting major gold deposits. The Birimian Supergroup, consisting of volcanic and sedimentary rocks, is particularly noted for its large-scale gold mineralization. The Birimian Supergroup consists of volcanic and sedimentary rocks that have been subjected to tectonic activity, making them favourable for the formation of mesothermal gold deposits. According to Griffis et al. [45], the Birimian rocks in Ghana are extensively folded and faulted, creating shear zones that act as conduits for hydrothermal fluids responsible for gold deposition. These shear zones, often intersected by regional-scale fault systems, are ideal traps for gold-bearing fluids, leading to the formation of rich gold deposits. Studies by Boamah & Koeberl [32] on the Bosumtwi impact structure and Osae et al. [17] on the Birimian rocks all in the Ashanti Region of Ghana confirm that gold deposits are associated with these formations, further emphasizing their importance in gold mining operations. The Tarkwaian Group, on the other hand, is composed mainly of clastic sedimentary rocks, including conglomerates and quartzites, which host significant paleoplacer gold deposits [19]. As noted by Hirdes and Nunoo [46], the Tarkwaian formations contain gold that was originally deposited in ancient riverbeds and subsequently concentrated by sedimentary processes [47].

Two significant epigenetic gold-forming occurrences have been documented in the world-class gold province of southwest Ghana. The Tarkwa paleoplacers, which are of world-class status, were the result of a pre-Tarkwaian event, while the deformation following the Birimian and Tarkwaian periods, associated with the Eburnean orogeny, led to the formation of orogenic gold deposits ranging from world-class (e.g. Prestea) to giant (e.g. Obuasi). These deposits have contributed to the region's renown for over 2500 years [48]. These paleoplacer deposits are some of Ghana's most productive sources of gold, particularly in regions like the Tarkwa and Damang mines. These regions have attracted major mining companies, such as Newmont Gold Ghana Limited and AngloGold Ashanti, underscoring the importance of these geological formations to the country's gold mining industry [20].

The works of Dzigbodzi-Adjimah and Asamoah [49], shows the geology of gold deposits of Prestea gold belts to be mainly of Birimian rocks consisting of Quartz Vein Type (QVT) which carries higher Au grades and Disseminated Sulphide Type (DST) which is mostly found on sheared or crushed near fissure Zones. Also, at Damang (the southwestern part of Ghana) shows gold deposits of paleoplacer in the Bankets series conglomerates of the Tarkwaian system [50].

The mineralized systems in southwest Ghana are commonly categorized as either orogenic lode-gold deposits (e.g. Obuasi) or paleoplacer gold deposits (e.g. Tarkwa) [22,51,52]. The Obuasi gold deposit is located within the Paleoproterozoic Birimian terranes of West Africa with 62 Moz of gold. The super giant Obuasi gold deposit is hosted in the Paleoproterozoic Kumasi Group sedimentary rocks composed of carbonaceous phyllites, slates, psammites, and volcaniclastic rocks intruded by different generations of felsic dikes and granites [48].

Localized studies within Ghana have further emphasized the importance of structural geology in controlling gold mineralization. For instance, the Ashanti Gold Belt, which includes the Obuasi and Prestea mining districts, is one of the richest gold-bearing regions in the country [53]. The mesothermal gold deposits in this belt are closely associated with shear zones, which have been extensively studied for their role in controlling the movement and deposition of gold [54]. The work of Dzigbodi-Adjimah and Nana Asamoah [55] also highlights the tectonic controls on gold mineralization in the Ashanti Belt, showing how faulting and folding have created favourable conditions for gold-bearing fluids to accumulate.

In addition to these primary geological formations, Ghana's gold mineralization is influenced by the broader tectonic framework of the West African Craton. The craton is characterized by a stable continental core surrounded by mobile belts, which have undergone significant tectonic deformation over geological time scales. This tectonic activity has played a critical role in remobilizing and concentrating gold within the Birimian and Tarkwaian formations [12].

Local mining communities have long recognized the gold potential in these formations, engaging in both small-scale and large-scale mining activities [56]. For instance, artisanal mining, commonly known as "galamsey," is a widespread practice in many gold-bearing areas. These miners rely on traditional knowledge of the land, passed down through generations, to identify areas rich in gold. However, these small-scale operations often lack the technological resources to optimize exploration and extraction processes, leading to inefficient practices and significant environmental degradation [57]. The need for integrating local knowledge with modern geospatial techniques is therefore critical for improving both the efficiency and sustainability of gold mining in Ghana [58].

Geospatial techniques, such as Geographic Information Systems (GIS) and remote sensing, have become indispensable tools in assessing the spatial distribution of gold-bearing rocks and mining activities. GIS technology enables the visualization and analysis of spatial data, providing insights into the geological formations that host gold deposits [25,59]. Several studies have demonstrated the effectiveness of GIS in identifying and mapping gold-bearing areas.

These methods provide very precise delineation of the mineralized zones and have turned what was a general discovery of gold deposits into very specific spatial information about areas that could be mined [60]. In conjunction with geophysical surveys, geochemical analysis, and field mapping, researchers can construct comprehensive models that predict gold deposits with a very high degree of accuracy [61].

In Africa, the continent's gold-rich regions, including South Africa's Witwatersrand Basin and the West African Craton, have benefited from the application of geospatial techniques. The use of geospatial techniques in gold exploration has been growing, especially in countries with significant mining potential. In Australia, the Yandal Gold Province is a prominent example where geospatial techniques such as GIS-based predictive modelling has been used to delineate potential gold-bearing areas within structurally complex terrains [5]. Similarly, in Canada, the Abitibi Greenstone Belt has been extensively explored using Remote Sensing and GIS, with significant success in identifying new gold zones [62]. The integration of geospatial data with traditional geological mapping has allowed for more targeted exploration efforts, thereby reducing the costs and environmental impact associated with gold mining. In Mali, for instance, GIS and Remote Sensing have been employed to assess gold potential within the Birimian Greenstone Belt [63]. These tools allow for the integration of geological, geophysical, and geochemical data to create models that highlight high-potential areas for further exploration. Similarly, in Burkina Faso, studies have shown the successful application of predictive models that use geospatial techniques to assess gold mineralization potential within shear zones [64].

The application of geospatial techniques in Ghana's gold exploration has significantly improved the efficiency of mineral exploration. Remote sensing effectively identifies surface anomalies like iron oxides and hydroxyl-bearing minerals associated with gold mineralization. In the Southern Kibi-Winneba Belt of Ghana, Forson et al. [65] used remote sensing and geophysical data to map alteration zones linked to gold deposits, highlighting areas for potential exploration. Similarly, Forson & Amponsah [66] applied spatial techniques and geophysical data in the Josephine prospecting license area, Northwestern Ghana, to predict gold mineralization zones, identifying key surface anomalies that aid exploration. Both studies demonstrate the crucial role of remote sensing in enhancing gold exploration in Ghana, leveraging local geological features and conditions. Moomen et al. [29] used GIS to evaluate the spatial distribution of gold-bearing formations in northern Ghana, providing valuable data for mining companies and policymakers. Lin et al. [30] also applied geospatial techniques to assess the environmental impacts of gold mining, highlighting the role of these technologies in sustainable resource management. According to Kazapoe et al. [67], multispectral and hyperspectral imagery have proven effective in mapping alteration zones indicative of gold-bearing hydrothermal systems in Ghana. GIS has also played a critical role in predictive modelling of gold deposits. By integrating geological, geophysical, and geochemical datasets, GIS allows for the identification of patterns that may indicate the presence of gold. Karikari [68] used GIS-based predictive mapping techniques to assess gold potential in the Lawra Belt of northwestern Ghana. The study integrated data on known gold occurrences, fault systems, and geophysical anomalies to create models that delineated zones with high exploration potential. In the Birim North District, a study conducted by GPMU [69] demonstrated the successful integration of satellite imagery with geological data to create predictive models for gold exploration. The use of geospatial techniques allowed the researchers to identify key gold-bearing zones, which were later validated through field investigations. The application of geospatial techniques in gold mining allows for a more efficient and targeted exploration of mineral resources. By integrating geological data with spatial analysis, researchers can better understand the distribution of gold-bearing rocks and optimize mining operations [27]. The ability to identify areas with high potential for gold mineralization helps mining companies focus their efforts on the most promising locations, reducing exploration costs and minimizing environmental impacts. This approach is particularly useful in regions like Ghana, where gold deposits are concentrated in specific geological formations, such as the Birimian and Tarkwaian rocks.

Gold mining in Ghana, especially within the Birimian and Tarkwaian rock formations, presents both environmental and socio-economic challenges. Activities by companies like Newmont Gold Ghana Limited and AngloGold Ashanti are mostly accused of land degradation, deforestation, and water contamination, impacting local livelihoods and biodiversity [58]. Communities face loss of agricultural land, and tensions arise from displacement and inadequate compensation, particularly in areas like Tarkwa, a hub for large-scale mining [70]. Hilson et al. [71] suggest that mining companies should enhance their corporate social responsibility (CSR) efforts to better address these environmental and social impacts.

**Fig. 2:**
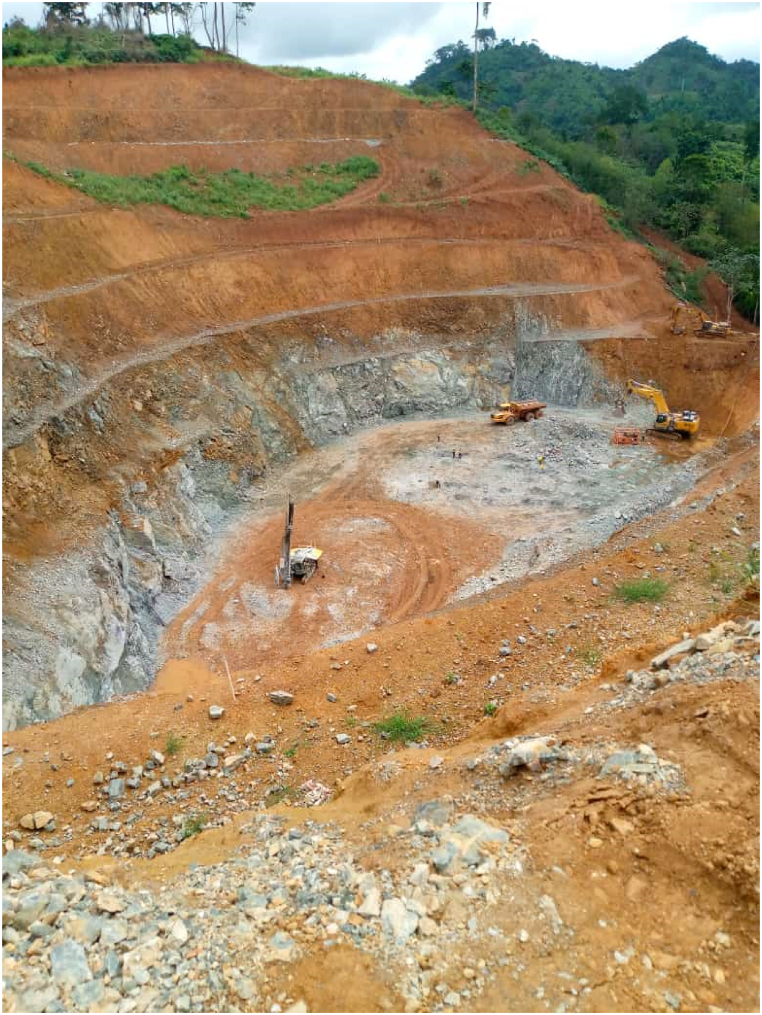
Field photograph of Chirano Gold Mines Ltd – Mamnao North Pit, located in the Western North Region. The mine operates on both Birimian and Tarkwaian rock formations, which are known for their gold-bearing potential.

**Fig. 3:**
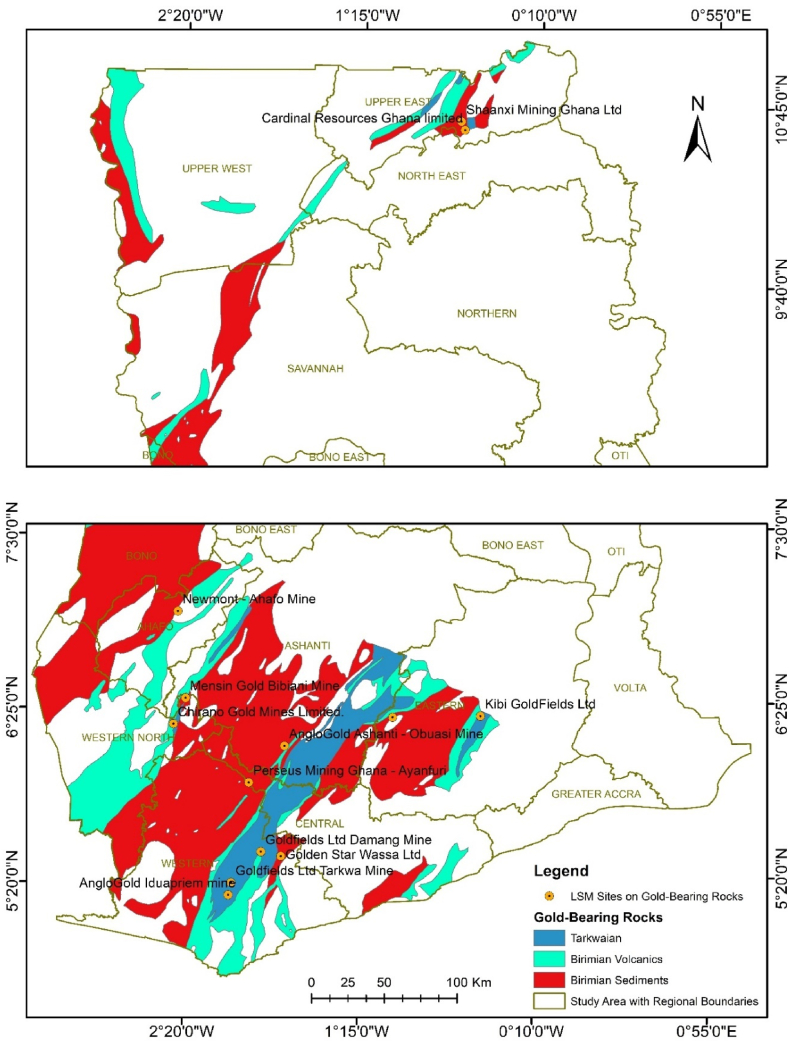
Pictorial representation of large-scale mining (LSM) activities within gold-bearing rock formations in the study area, derived from Google Earth Pro imagery, highlighting the spatial correlation between mining operations and gold-rich geological formations.

Fig. 2 presents a field photograph of Chirano Gold Mines Ltd – Mamnao North Pit, located in the Western North Region. The mine operates within Birimian and Tarkwaian rock formations, which are well-known for their gold-bearing potential. Fig. 3 provides a pictorial representation of large-scale mining (LSM) activities within gold-bearing rock formations in the study area, derived from Google Earth Pro imagery. This visualization highlights the spatial correlation between mining operations and gold-rich geological formations, reinforcing the significance of these rock types in gold mineralization.

While mining boosts the economy, sustainable practices - supported by geospatial data and local input are vital to protect the environment and ensure fair outcomes for communities. The integration of geospatial techniques with geological studies provides a comprehensive framework for understanding the relationship between Ghana's geology and its gold mining industry.

While previous studies have provided valuable insights into gold-bearing rocks in Ghana, they have primarily focused on specific regions or smaller localities, such as Tarkwa, Prestea, or the Ashanti Region. The current body of scholarly work is deficient in its determination of the spatial distribution of gold-bearing rocks across the entire country. Consequently, the objective of this study is to deliver a comprehensive analysis of the spatial distribution of gold-bearing rocks within Ghana, systematically classifying them according to regional delineations.

### 2.3 Datasets

The geological data of Ghana was obtained from the Geological Survey Department's (now Ghana Geological Survey Authority, GGSA) website (https://ggsa.gov.gh/*)*. The data was in a shapefile format and covered the entire study area.

### 2.4 Gold mineralization in Ghana

Birimian and Tarkwaian rocks have gold deposits distributed within them. Even though many rock types form Ghana's geology, numerous studies have proven that Birimian and Tarkwaian rocks contain gold deposits. Piper & Lomax [11] and Leube et al. [12] revealed that gold mineralization was found within the Birimian and Tarkwaian rocks. Years later, other studies proved same results that the gold-bearing rocks are the Birimian and Tarkwaian [[13], [14], [15], [16],^19^,^20^,^23^].

### 2.5 Geospatial process

ArcMap 10.8 was used to run the geospatial analysis. After ArcMap was launched, the Geology of Ghana shapefile was added. The attribute table was opened and explored to know the various fields. From the attribute table, one of the fields had information on the ERA Series. The ERA series had 15 rock types under it, and these cover the entire Ghana. According to Leube et al. [12], Osae et al. [17], Théveniaut & Clarke [23]; Smith et al. [19], and Webrah Kazapoe et al. [20], the gold-bearing rocks in Ghana Geology are Tarkwaian, Birimian Sediments, and Birimian Volcanics. Hence, these ERA Series rocks were selected.

**Fig. 4:**
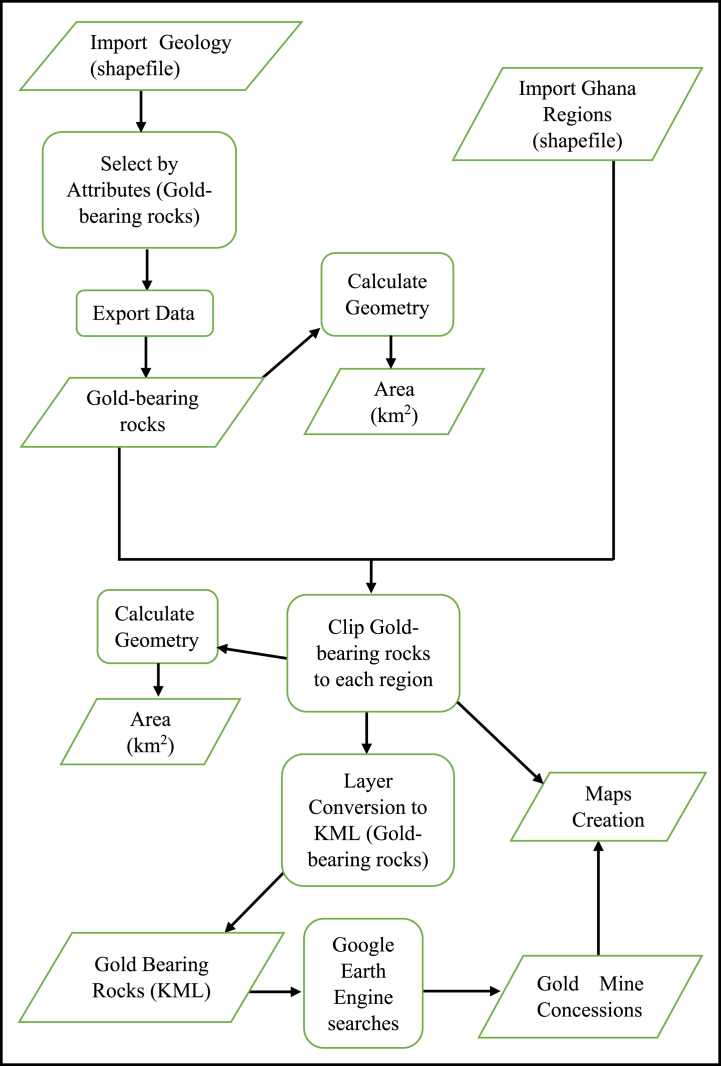
Flowchart of the geospatial process.

Fig. 4 shows the flowchart of the geospatial process for this study.

#### 2.5.1 Selecting the gold-bearing rocks

The selection of gold-bearing rocks was performed using the following method:

a) Opening the Attribute Table: The attribute table of the "geology" layer was accessed to begin the selection process.
b) Selecting by Attributes: The "Select By Attributes" tool was utilized to identify the specific geological eras associated with gold deposits. The following steps were taken:
  • In the "Select By Attributes" dialog box, "ERA_SERIES" was double-clicked to add it to the query builder.
  • The " = " operator was selected.
  • The "Get Unique" button was clicked to display unique values in the "ERA_SERIES" field.
  • "Tarkwaian" was selected from the list of unique values.
  • An OR condition was added to include additional relevant geological eras. This process was repeated for "Birimian Sediments" and "Birimian Volcanics"
c) Formulating the Query: The final selection query was constructed as follows:

"ERA_SERIES" = 'Tarkwaian' OR "ERA_SERIES" = 'Birimian Sediments' OR "ERA_SERIES" = 'Birimian Volcanics'

d) **Applying the Query:** The query was applied to the attribute table, resulting in the selection of rocks classified under the Tarkwaian, Birimian Sediments, and Birimian Volcanics eras.
e) **Exporting Selected Features:** The selected features were exported by right-clicking on the "geology" layer, selecting "Data", and then "Export Data". The output was saved under the name "Gold_Bearing_Rocks" in the designated directory.

This methodological approach ensured the accurate identification and selection of gold-bearing rock formations within the study area.

Subsequently, the gold-bearing rocks were clipped to the regions where these rocks occupied using the Clip Tool. The area covered by the gold-bearing rocks for each region were calculated to draw statistical inferences.

#### 2.5.2 Area computation

A new field was added in the Attribute Table for the 'Gold_Bearing_Rocks' layer for one region having the gold-bearing rocks. The header of this field was named “Area_sq_km” representing area in square kilometers. The column of this added filed was selected and the Calculate Geometry tool is used to populate the areas for the rows. The area unit selected was square kilometers.

The same procedure was repeated to compute the area for the remaining regions, and the other geological compositions for the entire country.

#### 2.5.3 Ground truth verification

**Fig. 5:**
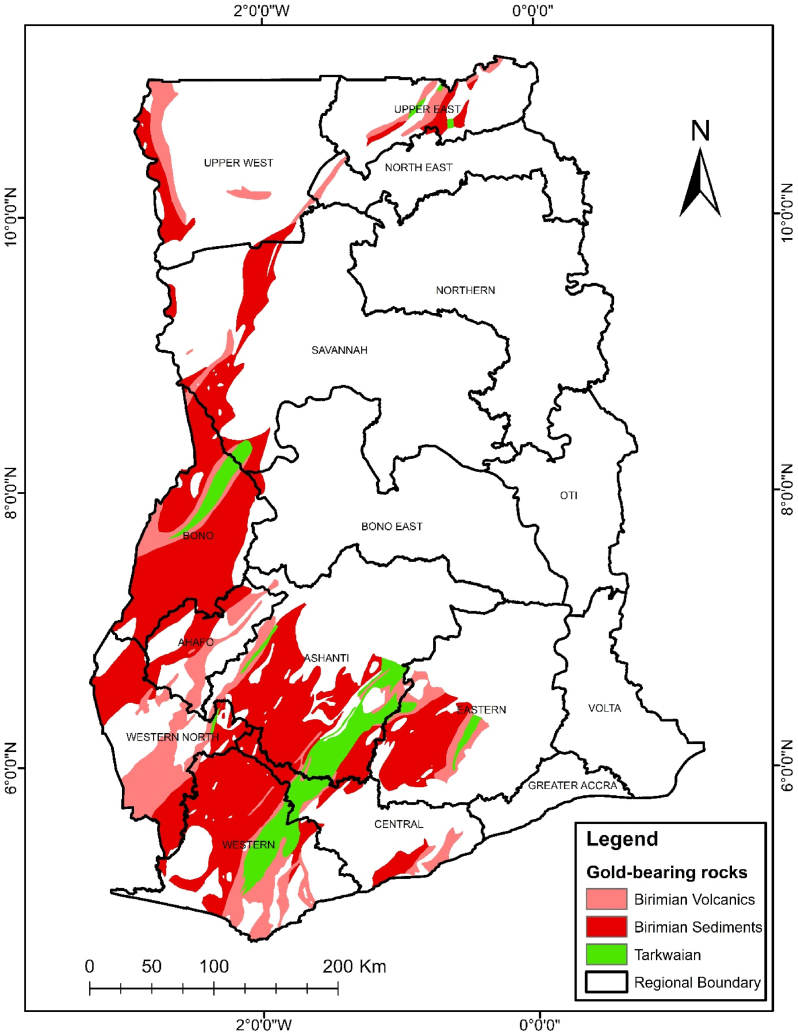
Map of the spatial distribution of gold-bearing rocks within Ghana.

The Gold Bearing Rocks layer was converted to KML using the Conversion tool in ArcToolbox. The KML file was opened in Google Earth Engine. In Google Earth Engine, Ghana's various gold mining companies were searched, and their locations were superimposed on the Gold Bearing Rocks. All the gold mining companies found were situated on the gold-bearing rock (Fig. 5). An onsite visit was made to the majority of the Gold Mining Companies, namely, Goldfields Limited - Tarkwa Mines, Goldfields Limited - Damang Mines, AngloGold Ashanti - Iduapriem Mine, Newmont Ridge Limited - Akyem Mine, Newmont Gold Ghana Limited - Ahafo South, Perseus Mining Limited, Cardinal Ghana Resource Limited, Chirano Gold Mines Limited, Asanko Mines, AngloGold Ashanti - Obuasi Mines, and Adamus Resources Limited.

#### 2.5.4 Tables and map creation

**Fig. 6:**
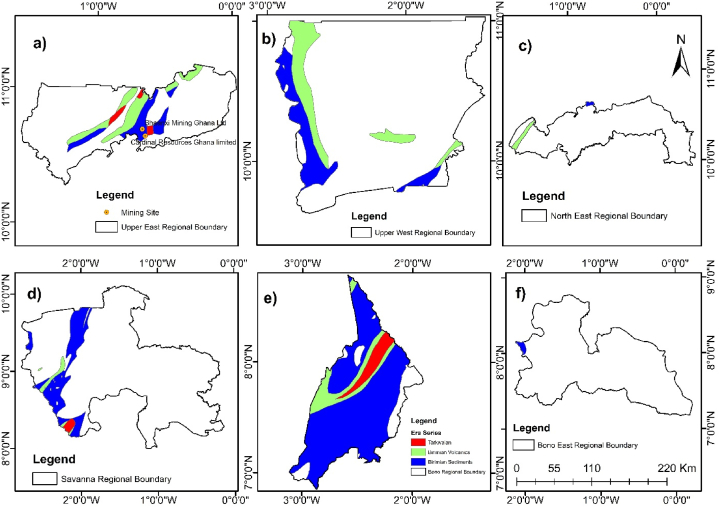
Spatial Distribution of gold-bearing rocks in Ghana with mining sites according to regions a) Upper East Region b) Upper West Region c) North East Region d) Savanna Region e) Bono Region f) Bono East Region.

**Fig. 7:**
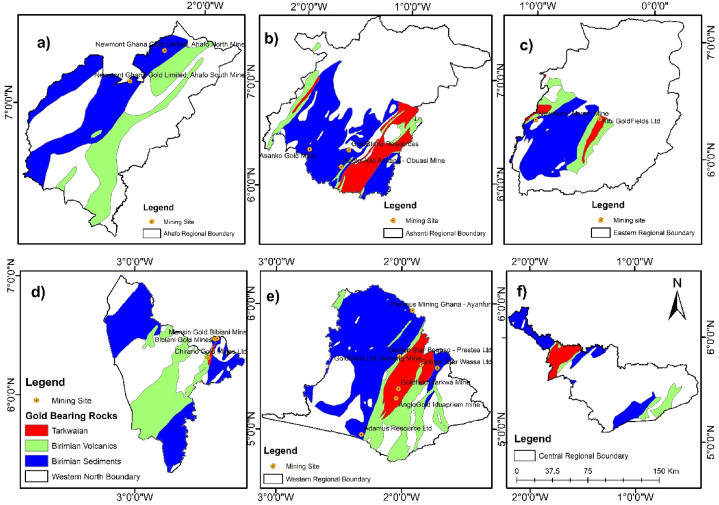
Spatial Distribution of gold-bearing rocks in Ghana with mining sites according to regions a) Ahafo Region b) Ashanti Region c) Eastern Region d) Western North Region e) Western Region f) Central Region.

**Fig. 8:**
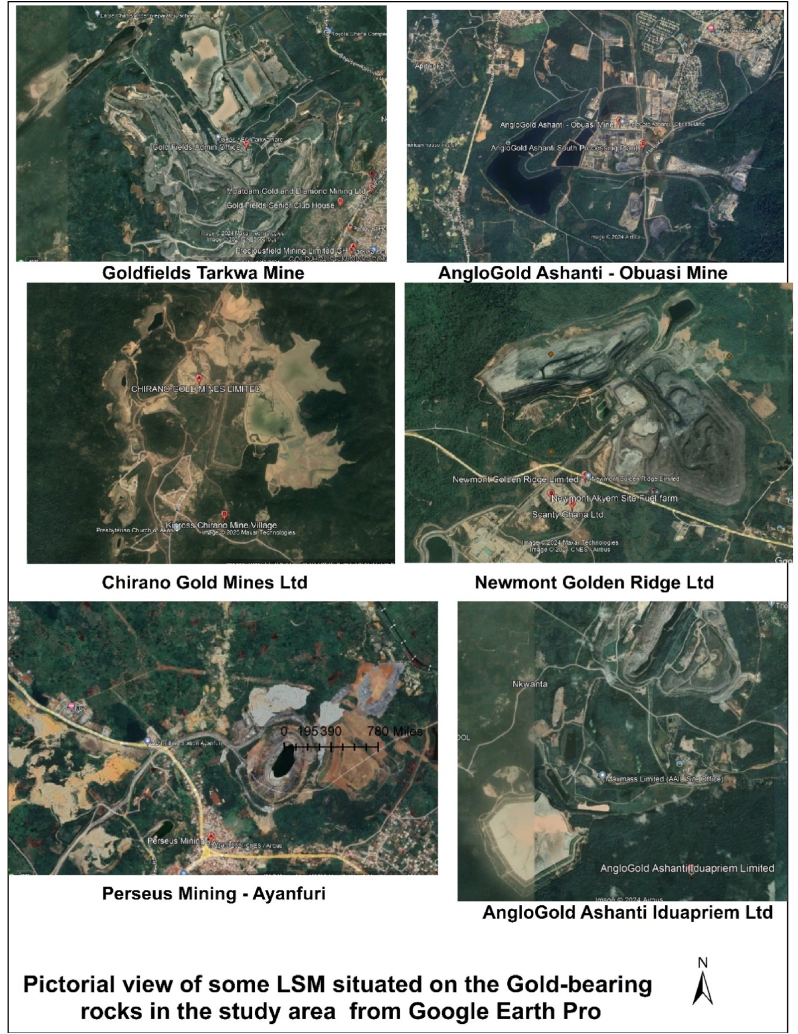
Map of mining concessions located within gold-bearing rocks spatial distributed within Ghana (Google Earth Pro).

The final maps were created by adding map elements. The maps generated were Ghana's Geology, Gold Bearing Rocks, and Gold Mining Companies located within the Gold Bearing Rocks; and exported as a JPEG file (Fig. 5, Fig. 6(a–f), Fig. 7(a–f), and Fig. 8). The attribute table of each layer was opened, and the data was copied. Microsoft Excel was used to compute the percentages of each rock-type area.

## 3 Results

The gold-bearing rocks spatial distribution in Ghana is displayed in Fig. 5. These rocks are the Tarkwaian, and the Birimian Supergroups. Majority of them are clustered in the southwestern part of Ghana.

To determine the spatial distribution of these rocks across the regions of Ghana, Fig. 6(a–f) and Fig. 7(a–f) shows the regions where the gold-bearing rocks can be found and their distribution spatially.

The gold-bearing rocks were found in twelve regions of Ghana with the Bono East region having the least of these rocks.

Subsequently, the area (in Km^2^ or %) occupied by the three gold-bearing rocks: Tarkwaian, Birimian Sediments, and Birimian Volcanics are represented in Table 1.

**Table 1:** Regional area of gold-bearing rocks distribution in Ghana.

Regions	Rocks Bearing Gold Area (km)	Area (%)
Upper East	1523.84	2.74
Upper West	2898.86	5.22
North East	226.60	0.41
Savanna	4019.59	7.24
Bono	10410.27	18.74
Bono East	137.75	0.25
Ahafo	2831.47	5.10
Western - North	6360.87	11.45
Western	9735.60	17.53
Ashanti	9957.91	17.93
Eastern	4707.11	8.48
Central	2730.12	4.92
**Total**	**55540.00**	**100.00**

The Bono region has the largest area of these rocks, followed by the Ashanti, Western region and then the Western North regions respectively (Table 1). These four regions altogether host 65.65 % of the gold bearing rocks.

It was observed from Fig. 6(e) that even though the Bono region has the largest size of the gold-bearing rocks (10410.27 km^2^), there is no notable large-scale mining (LSM) company operating on these formations.

Additionally, the Bono Region composition is 90.31 % gold-bearing rocks, which are all the three rocks (i.e Tarkwaian, Birimian Sediments, and Birimian Volcanics). Majority of these rocks are Birimian Sediments as shown in Fig. 6(e).

The Western region has the largest number of large-scale mining companies located on the gold-bearing rocks as displayed in Fig. 7(e). AngloGold Ashanti - Iduapriem, Goldfields Tarkwa, and Golden Star Ltd mines are operating on Tarkwaian rocks.

Ground truth verification using Google Earth Pro and onsite visits validation of the spatial correlation between gold mining companies and gold-bearing rock formations, highlighting geology's role in mining activities. Fig. 8 shows the spatial distribution of the gold-bearing rocks with google Earth Engine images of the mining concessions.

Ghana's geology is composed of 23.34 % gold-bearing rocks (Appendix 2), among which Birimian Sediments occupy 15.40 % (Appendix 1). Of the gold-bearing rocks, the Birimaian Sediments has the largest composition of 65.99 %, and Tarkwaian are the least with 10.29 % (Appendix 3).

## 4 Discussion

The research presents a profound exploration of the intricate relationship between the country's geological composition and its stature as a leading gold producer in Africa. Ghana's geological landscape, characterized by diverse rock formations, plays a pivotal role in shaping the distribution of gold deposits across the country. The study focuses on key formations such as the Birimian Volcanics, Birimian Sediments, and Tarkwaian rocks, which have been identified in Fig. 5 as significant hosts of gold mineralization across the entire country. Through the lens of Geographic Information Systems (GIS) and spatial analysis, the research unveils the spatial patterns of gold mineralization dominantly located in the southwestern part of Ghana, which have attracted major mining companies like Newmont Gold Ghana Limited, Tarkwa Gold Fields Ltd and AngloGold Ashanti, underscoring the geological link between specific rock types and gold-rich zones.

Geospatial techniques, particularly Geographic Information Systems (GIS), serve as indispensable tools in analyzing and visualizing the spatial distribution of gold-bearing rocks in Ghana. Ghana's Era series geology has 15 rock types, offering a visual representation of the geological formations that host gold deposits. Notably, the Obosum & Oti Beds represent the largest formation, covering 79,466.10 km^2^ (33.29 %) of Ghana's geological area (Appendix 1). Although the Obosum & Oti rocks occupy the largest portion of Ghana's geology, studies have not yet revealed any viable mineral reserve. Spatial analysis reveals that 23.34 % (55,723.62 km^2^) of Ghana's geology consists of gold-bearing rocks (Appendix 3), with Birimian Sediments covering the largest area, 36,771.5 km^2^ (65.99 %) of the gold-bearing rocks.

Among the rocks that form Ghana's geology are the Tarkwaian, Birimian Sediments and Birimian Volcanics. Tarkwaian and Birimian rocks are the custodians of gold mineralization in Ghana before time memorial. Gold mining operations have taken place in many regions in Ghana proven to contain these rocks. Presently, gold mining activities are still taken place on Tarkwaian and Birimian rocks in many parts of Ghana which are consistent with studies of Leube et al. [12]; G. Hilson, [58]; Yidana et al., [70]; Kumi et al., [72]. These rocks have boosted Ghana's economy significantly looking through the lenses of the mining industry [72]. Tarkwaian and Birimian rocks are central to gold mining in Ghana's Western Region, with major operations like Goldfields Ltd.'s Tarkwa and Damang Mines, and AngloGold Ashanti's Iduapriem Mine situated on Tarkwaian rocks, reflecting their gold-bearing potential. Adamus Resource Limited's operations on Birimian Sediments further emphasize the significance of Birimian formations in gold mineralization (Fig. 5). In the Eastern Region, Newmont Golden Ridge (Akyem Mine) and Kibi Goldfields Ltd are located on Birimian Sediments, Birimian Volcanics, and Tarkwaian rocks, demonstrating a strong correlation between geological formations and mining activities. Even though there are 4707.11 km^2^ (8.48 %) of the gold-bearing rocks in the Eastern Region, only these two large scale mining companies are operational there. Investors in the large-scale mining sector can invest to establish other mining companies in the Eastern region since only few square kilometers are occupied by the two large scale mining companies. Furthermore, government can ensure that illegal mining activities in this region is reduced to the barest minimum through registration of concessions and policy enforcement.

In the Ashanti Region, AngloGold Ashanti-Obuasi Mine, Asanko Mines, and Goldstone resources operate on Birimian Sediments, confirming what previous studies have identified as gold belts (Fig. 6(a–f) and Fig. 7(a–f)). Hence, the Birimian and Tarkwaian rocks were named gold-bearing rocks. The precise location of these mines on the Birimian and Tarkwaian rocks justifies the literature claims [[11], [12], [13], [14], [15], [16], [17], [18], [19], [20]] that Birimian and Tarkwaian rocks are gold-bearing rocks.

These formations align with structural and tectonic controls, as seen in other gold-rich provinces worldwide. For example, Chen et al. [1] emphasize the importance of carbonate-sulfide vein-type deposits within the Jiaodong Gold Province, China, which is similar to the mesothermal quartz vein-hosted deposits found in Ghana's Birimains, were shaped by significant shear zones and fault structures.

The Paleoproterozoic Eburnean Orogeny is fundamentally connected to the origin of gold deposits in Ghana, influencing the types of lithologies we find [73]. This occurrence illustrates a broader trend in global gold mineralization, where various orogenic events shape the deposits' characteristics. Chen et al. [7] investigated the tectonic shifts from the Jurassic to the Cretaceous in Jiaodong, revealing how distinct geological factors contribute to gold formation. Heritier et al. [74] conducted similar investigations into hydrothermal gold systems found within the Kibaran metallogenic province located in the DR Congo, illustrating how the movement of deep fluids along fault lines mirrors the processes that influence Ghana's lode gold deposits.

Additionally, ground truth verification using Google Earth Pro and onsite visits confirms the spatial correlation between gold mining companies and gold-bearing rock formations, highlighting geology's role in mining activities. This process validates the accuracy of spatial data and the precise locations of mining companies, supporting the reliability of geospatial analysis in assessing Ghana geology's impact on gold mining. The gold-bearing rocks cut across twelve regions of Ghana out of sixteen. These are the Ashanti, Western, Eastern, Central, Upper East, Upper West, Bono, Ahafo, Savannah, North East, Western North, and a little outcrop in Bono East regions (Fig. 6(a–f) and Fig. 7(a–f)). This gives a possibility of gold mining activities happening in these twelve regions. Yet some of these regions do not have any large-scale mining company operating on it. From the Google Earth Pro verification and onsite visits, the Bono region does not have any large-scale mining company operating there, even though 90.31 % of the Bono region is composed of gold-bearing rocks. This region also has the least illegal gold mining activities occurrences. Hence, stakeholders in the mining sector can extend their gold exploration works in this region.

Most of these formations are primarily located in the southwestern areas of Ghana, which are critical for gold mining activities. The onsite and ground truth verification has proven this to be true and implies these twelve regions will be experiencing almost similar socio-economic and environmental conditions.

Subsequently, Google Earth Pro observations made revealed that the Central region has series of illegal mining concessions which has no known names within the gold-bearing rocks in the region (Fig. 7(f)). This also validates gold mineralization in the Tarkwaian and Birimian rocks. Additionally, the presence these large-scale gold mining companies; - Cardinal Ghana Resource Limited, and Shaanxi Mining Ghana Ltd (Fig. 8) in the Upper East region shows the expansion of gold exploration works in the Northern part of Ghana. The remaining regions in the Northern part which have gold-bearing rocks but currently have no mining company operating provide a basemap for further exploration in such regions.

By leveraging GIS, spatial analyses help identify areas with high potential for gold mineralization, aiding in the strategic planning of mining operations and exploration activities. Integrating multi-geospatial data sources, including geological maps, remote sensing data, and geophysical surveys, enhances the understanding of subsurface geology and facilitates targeted exploration efforts.

The application of geospatial techniques in this study allows for a comprehensive assessment of the types of rocks that bear gold and their geospatial distribution. By employing various GIS tools, the study team was able to visualize the locations of gold-bearing rocks and the extent of mining activities concerning these formations.

This approach not only enhances the understanding of the geological context of gold deposits but also provides valuable data for stakeholders, including investors, government agencies, and researchers, in making informed decisions regarding land use and resource management.

Furthermore, the study accentuates Ghana's geology's economic and environmental implications on gold mining. The application of geospatial techniques in mineral prospecting has become an essential methodological approach in modern exploration studies.

Our study aligns with the principles of mineral prospectivity mapping (MPM), which synthesizes various geoscientific data using Geographic Information Systems (GIS) to pinpoint mineral-laden zones with greater accuracy. As emphasized by Carranza [75,76], GIS-based exploration targeting enables the effective compilation, analysis, and visualization of spatial datasets, ensuring a more systematic assessment of geological factors controlling gold mineralization. The integration of knowledge-driven and data-driven modeling, as noted by Yousefi et al. [77] and Yousefi et al. [78], improves predictive efficacy through the incorporation of essential geological, geochemical, and geophysical data. Our approach, employing geospatial techniques for prospectivity analysis, is bolstered by recent progress in machine learning algorithms and mineral system modeling [79,80], thereby enhancing the integrity of GIS-based exploration. By leveraging these geospatial methods, we provide a more refined and objective assessment of gold mineralization potential in Ghana, thereby reducing exploration uncertainties and improving decision-making in mineral resource management. The concentration of mining activities within specific geological formations highlights the importance of considering geological factors in decision-making processes related to mining operations and sustainable development initiatives. Findings from this research are essential for effective land use planning and biodiversity conservation. By mapping gold-bearing rocks and mining sites, planners can allocate specific zones for mining, preventing conflicts with agriculture, forestry, and urban areas and promoting sustainable development. The gold mining industry is the stronghold of Ghana's economy optimizing the exploration and extraction processes through geospatial analysis, the sector can increase efficiency and profitability. This, in turn, can boost economic growth, create jobs, and generate revenue for the government. Local communities will also benefit when they get a clear understanding of where gold-bearing rocks are located. This will enhance transparent compensation and the development of mining communities.

In a wider view, mining activities in areas rich in gold-bearing rocks can lead to substantial environmental degradation, including land degradation, deforestation, soil erosion, and contamination of water bodies. The disruption of local ecosystems, loss of vegetation, and reduced biodiversity are critical environmental concerns that need to be addressed through sustainable mining practices. Due to the challenges mining has on the communities they mine, the mining companies should increase their contributions in the area of corporate social responsibility (CSR) especially in communities where the gold-bearing rocks are located. This must be taken seriously since many studies in sub-Saharan Africa show that only very little is done by mining companies in terms of CSR [71]. Economically, the presence of major towns on gold-bearing rocks poses challenges for expanding mining operations due to high compensation costs for land acquisition. For instance, Tarkwa and Bolgatanga municipalities in the western and upper-East regions respectively are located on Birimian Sediments and Birimian Volcanics formations, making it economically unviable to expand or establish new mining concessions in such developed areas.

Moreover, the study's emphasis on the importance of considering the location of gold-bearing rocks in land use planning and resource management underscores the need for sustainable practices in Ghana's mining sector. Diego et al. [81] confirm that gold mining is expanding globally, presenting various economic, social, and environmental challenges and opportunities. Economically, it boosts local economies and creates jobs but can also lead to displacement and conflict. Environmentally, it promotes technological advancements but often causes deforestation, soil degradation, and water contamination. Balancing these impacts is crucial for sustainable development. By unraveling the geological complexities that govern gold mineralization and mining activities, the research contributes to informed decision-making, sustainable development, and effective resource management in the country's rich geological landscape.

The study shows that Ghana's geology, particularly the distribution of Birimian Sediments, Birimian Volcanics, and Tarkwaian rocks, greatly impacts gold mining locations. Understanding this geological landscape is crucial for resource management and sustainable development. Local communities can benefit from continuous geological mapping to uncover new gold-bearing areas and the implementation of sustainable mining practices to protect the environment. Integrating geological data into land use planning will help balance mining and settlement development. Additionally, raising awareness about the importance of geological formations and the environmental impacts of mining will empower local communities to engage in resource management effectively.

Future studies should combine geological and socio-economic data to improve decision-making in the mining sector, and since this study is limited to gold mining companies’, other studies can focus on other minerals like diamond, bauxite, and manganese.

## 5 Conclusion

This study applied geospatial techniques to assess the impact of Ghana's geology on gold mining, highlighting the spatial distribution and significance of gold-bearing formations. The findings confirmed that gold mineralization is concentrated within Tarkwaian, Birimian Sediments, and Birimian Volcanics, which together cover 55,723 km^2^ (23.34 %) of Ghana's land area. The highest concentration of the gold-bearing rocks was observed in the Bono (18.74 %), Ashanti (17.93 %), Western (17.53 %), and Western North (11.45 %) regions. Notably, large-scale mining companies, including Newmont Gold Ghana Limited, AngloGold Ashanti, and Goldfields Ghana Ltd, predominantly operate within these formations, reaffirming the geological control on gold localization.

Ground truth verification using Google Earth Engine and field surveys further validated the strong correlation between mining activities and gold-bearing formations. Despite the extensive presence of gold-bearing rocks in the Bono Region (90.31 % of its geology), no significant large-scale mining operations exist there, presenting an opportunity for future exploration. Conversely, illegal mining activities were identified in regions like the Central Region, emphasizing the need for stricter regulatory enforcement and sustainable mining practices.

The study highlights the importance of integrating geological data into land-use planning to optimize resource management, reduce compensation costs for settlements on gold-bearing formations, and mitigate conflicts between mining and urban development. It likewise points out the necessity for enhanced corporate social responsibility (CSR) endeavors by mining corporations, especially within the communities that are impacted.

Future research should incorporate geophysical and geochemical analyses to refine mineral exploration models. Additionally, the assessment of the socio-environmental impacts associated with mining activities in gold-rich locales is imperative to guarantee the sustainable utilization of natural resources.

## CRediT authorship contribution statement

**Aaron Tettey Tetteh:** Writing – review & editing, Writing – original draft, Visualization, Validation, Supervision, Software, Resources, Project administration, Methodology, Investigation, Formal analysis, Data curation, Conceptualization. **Yakubu Issaka:** Writing – review & editing, Visualization, Validation, Supervision, Methodology, Investigation, Formal analysis. **Philomena Dodoo:** Writing – review & editing, Writing – original draft, Visualization, Validation, Methodology, Formal analysis. **Bernice Atugba:** Writing – original draft, Visualization, Validation, Resources, Methodology, Data curation. **Albert Tengnibuor:** Writing – original draft, Visualization, Validation, Methodology, Formal analysis.

## Data availability statement

Data will be made available on request. For requesting data, please write to the corresponding author.

## Ethical statement

Not applicable for this research as it does not involve animal or human subjects for data collection.

## Declaration of competing interest

The authors declare that they have no known competing financial interests or personal relationships that could have appeared to influence the work reported in this paper.

## Figures and Tables

**Table:** 

ERA_SERIES	Area (km^2^)	Area (%)
Birimian Volcanics	13216.90	5.54
Dahomeyan	64282.00	26.93
Birimian Sediments	36771.50	15.40
Tarkwaian	5735.22	2.40
Basal Sandstone	2480.03	1.04
Obosum & Oti Beds	79466.10	33.29
Upper Voltaian	21253.70	8.90
Buem	7204.24	3.02
Togo Series	3754.25	1.57
Buem Volcanics	524.15	0.22
Tertially	868.55	0.36
Eocene & Cretaceous	889.50	0.37
Recent	2057.00	0.86
Accraian (Mid-Devonian)	45.32	0.02
Sekondian (?Devonian)	150.40	0.06

**Total**	**238698.86**	**100.00**

**Table:** 

ERA_SERIES	Area (km^2^)	Area (%)
Birimian Volcanics	13216.9	23.72
Birimian Sediments	36771.5	65.99
Tarkwaian	5735.22	10.29

**Total**	**55723.62**	**100.00**

**Table:** 

Era series	Area (km^2^)	Area (%)
Total Gold Bearing Rocks	55723.62	23.34
Total Era series of Ghana's geology	455622.44	100.00
